# An *In Vivo* Comparative Evaluation of Dental Anxiety Level and Clinical Success Rate of Composite and Multicolored Compomers in 6 to 12 years of Children

**DOI:** 10.5005/jp-journals-10005-1562

**Published:** 2018

**Authors:** Shivayogi M Hugar, Divyata Kohli, Chandrashekhar M Badakar, Niraj S Gokhale, Prachi J Thakkar, Madhura V Mundada

**Affiliations:** 1-6 Department of Pedodontics and Preventive Dentistry, Kaher's KLE VK Institute of Dental Sciences, Belagavi, Karnataka, India

**Keywords:** Anxiety, Children, Coloured compomers, Composites

## Abstract

**Background:**

Pediatric dentistry is not just about treating the tooth, but it also involves giving an overall comprehensive treatment to the child. Children like different colors and when the child is allowed to select the color of the restoration, it will positively motivate the child to accept dental treatment.

**Aim:**

The aim of our study was to evaluate and compare the clinical success rate of composite and multicolored compomer restorations and dental anxiety level in children.

**Materials and methods:**

A total of 60 samples equally divided into two study groups by of split-mouth design. In the control group, subjects received composites and in experimental group, they received colored compomers. The dental behavior was assessed using the Frankl behavior rating scale for both the groups. Dental anxiety was checked in the patients using visual analogue scale (VAS) before and after the treatment for both the groups. Children were recalled for follow up at 1, 3 and 6 months to evaluate clinical success rate amongst control and experimental group and results were subjected to statistical analysis.

**Results:**

Colored compomer proved to reduce the anxiety in the child and had a better behavioral response and positive attitude. Both restorative materials had comparable clinical success rates.

**Conclusion:**

At 6 months follow-up evaluation colored compomer restorative material showing promising with similar properties like that of composites with the added advantage of multicolors and can be considered as the new restorative material in the child dentistry.

**Clinical significance:**

Colored compomers are known to be excellent alternative restorative materials for restoration of teeth in children as they aid in behavior modification and good compliance from the patient.

**How to cite this article:**

Hugar SM, Kohli D, Badakar CM, Gokhale NS, Thakkar PJ, Mundada MV. An *In Vivo* Comparative Evaluation of Dental Anxiety Level and Clinical Success Rate of Composite and Multicolored Compomers in 6 to 12 years of Children. International Journal of Clinical Pediatric Dentistry, 2018;11(6):483-489.

## INTRODUCTION

Pediatric dentistry is not just about treating the tooth, but it also involves giving an overall comprehensive treatment to the child. As we all know that restorative dentistry is considered a failure if the child departs in tears so, as pediatric dentists, it is of utmost importance for us to understand the deep-seated apprehensions of a child and overcome them with various behavior modification techniques.

Early diagnosis and treatment of carious lesions in children are helpful for the upholding of oral health. Despite a general cut in caries rate, about 30% of all carious lesions apparent in 6-year-old children have not been treated with restoration due to very poor home care compliance and the fact that many of them are afraid to visit a dentist. Every child is unique in his own way. It is difficult to motivate these children to receive effective restorative therapy. Children are motivated to select a restorative material of their preference as some children like different colored restorative materials, and some may like white colored restorative material to look same like a tooth so that they can be positively involved and receive treatment. Color influences a child's life in ways which we cannot discern. In children who are uneasy and who simply refuse treatment, we can allow them to select multicolored restorative materials.^1^ Children are usually very pompous of their new fillings. This encourages maintenance of the restorations, which in turn significantly improves general oral hygiene and also instills a positive attitude in them towards the treatment.

In recent times, new materials have come to market even though composites being the ideal tooth material for permanent. A modified glass ionomer, now accessible called compomer, is a combination of composite resin and glass-ionomer cement. They have improved properties of traditional glass ionomers and also have material properties quite similar to composite resin. In last one decade, colored compomers have been available in the market for the restoration of primary teeth. When compared to conventional composites, they contain a minute quantity of glitter particles which produce a color effect ranging from innate tooth color with sparkle to pink, blue, and green. The filler content is alike to that of conventional compomers.^1,2^

There were no previous studies in India to evaluate and compare the acceptance of dental treatment and the change in the behavior of children receiving restorations with composites and colored compomers. This study was designed to evaluate and compare the clinical success, dental anxiety level in children using composites and multicolored compomers in children of 6–12 years.

## MATERIALS AND METHODS

In totality, 60 samples were selected reporting to the outpatient department of the Department of Pedodontics and Preventive Dentistry, Belagavi. Study samples were divided into two study groups namely control group (group A) and experimental group (group B) by lottery method, with 30 samples in each group. The details of the procedure were fully explained to the parents, and required permissions were obtained from the institutional review board, parents and the child.

The required children for the study were selected as per the selection criteria. The inclusion criteria used for the study were children with mixed dentition (6– 12 years of age) who have not visited the dental clinic in the past, shallow or moderate occlusal caries in first permanent molars children having at least 1–2 decayed teeth. The exclusion criteria considered were patients with high caries rate, proximal caries, patients wearing appliances children with special healthcare needs.

### Sample Size

The required sample size was calculated as follows:

**Figure:**
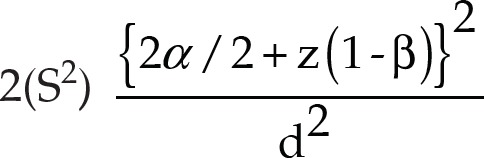


where *α* = 5%, z*α* =1.96 and *β* = 80%, z(1-*β*) = 0.824

where *α* = type 1 error, 1-*β* = power of the study, S = standard deviation, d = mean difference.

### Procedure

After comfortably seating the selected children on the dental chair, the step-by-step procedure was explained to the parents as well as the children. After rubber dam isolation, fluoride-free pumice prophylaxis was done on the concerned tooth. The tooth preparation was carried out as per the principles of minimum invasive dentistry. A bonding system (Futurabond NR, Voco) was used according to the manufacturer's instructions in both groups. Group A (control group) received composite material (3M ESPE, St. Paul, Minn., USA) and group B (experimental group) received colored compomer (Twinky star, Voco, Cuxhaven, Germany). Articulating paper was used to check occlusal irregularities. The immediate baseline evaluation was done by an experienced examiner (Pediatric dentist, who is blind to the study). The children were given post-operative instructions. The dental behavior was assessed using Frankl behavior rating scale,^3^ and dental anxiety was checked using visual analog scale (VAS)^4^ before and after the treatment by the same trained examiner to overcome operator bias.

The subjects were recalled for the follow up at 1, 3 and 6 months for the evaluation of the retention using the US public health service modified ryge criteria^5^ (Figs 1A to F).^2^ The collected data were entered into a master chart and subjected to statistical analysis using SPSS version 20.0 (Chicago, USA).

## RESULTS

In both the groups, on a comparison of preoperative and postoperative scores for dental anxiety using paired t-test, significant (*p* = 0.000) difference was seen. The difference between the pre- and post-values was more in group B (experimental group) as compared to group A (control group) indicating a significant decrease of dental anxiety posttreatment in group B (experimental group) (Table 1).

**Graph 1:**
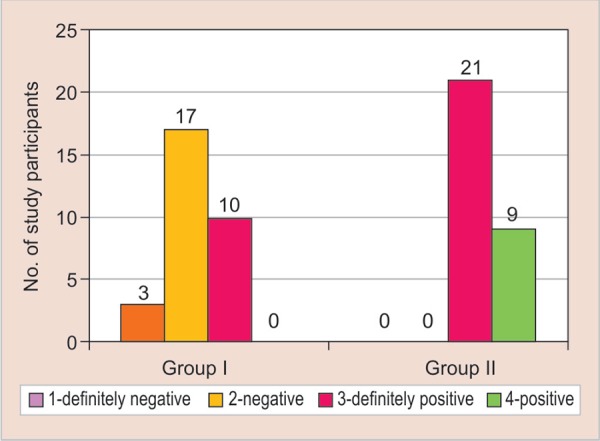
Graph showing the comparison of Frankl behavior rating for the conventional composite material in control group (group A) and colored compomer material in experimental group (group B)

On applying the chi-square test for Frankl behavior rating scale, the *p* value was found to be significant (*p* = 0.000). When unpaired t-test was applied to compare the two means, it was highly significant (*p* = 0.000, t = −13) with 35% showing a positive behavior and 15% showing a positive behavior for group B (experimental group) (Graph 1).

**Graph 2:**
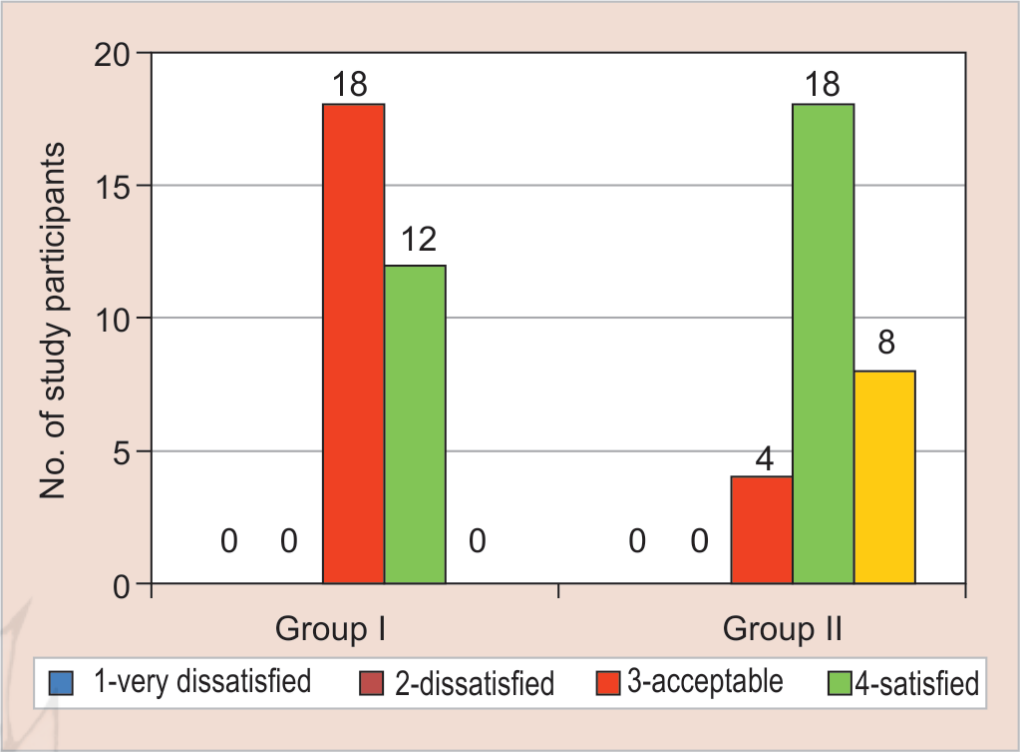
Graph showing the parental perception about change in behavior of the child towards dental treatment, for the conventional composite material in control group (group A) and colored compomer material in experimental group (group B).

**Graph 3:**
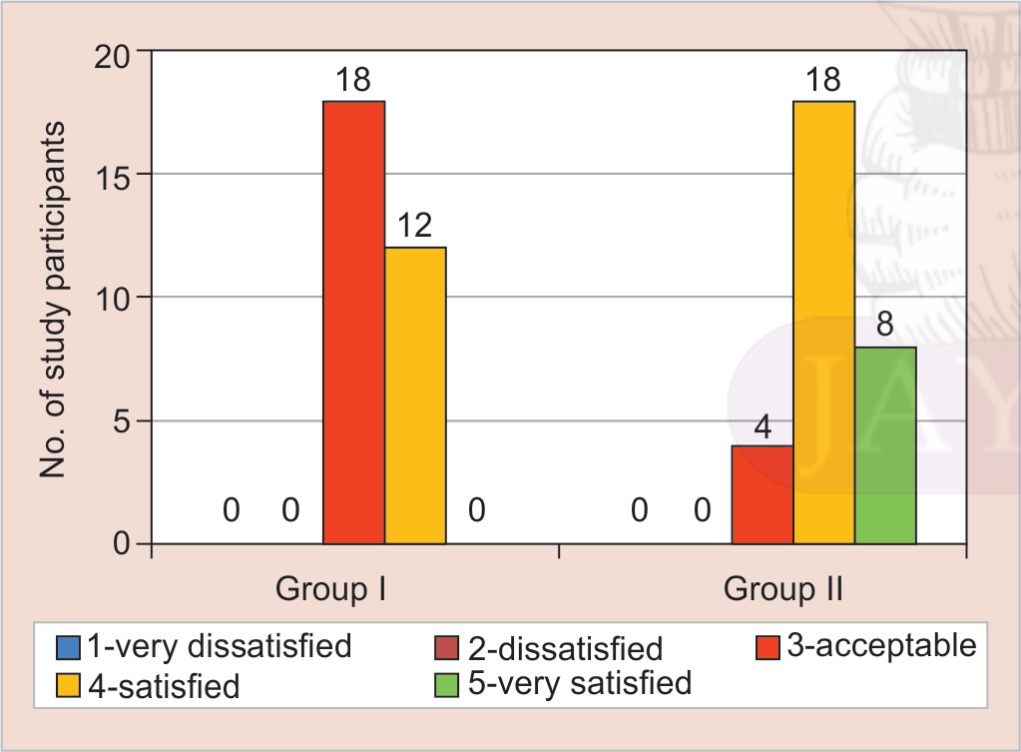
Parental perception about change in behavior of the child towards color of the restoration for the conventional composite material in control group (group A) and colored compomer material in experimental group (group B)

**Graph 4:**
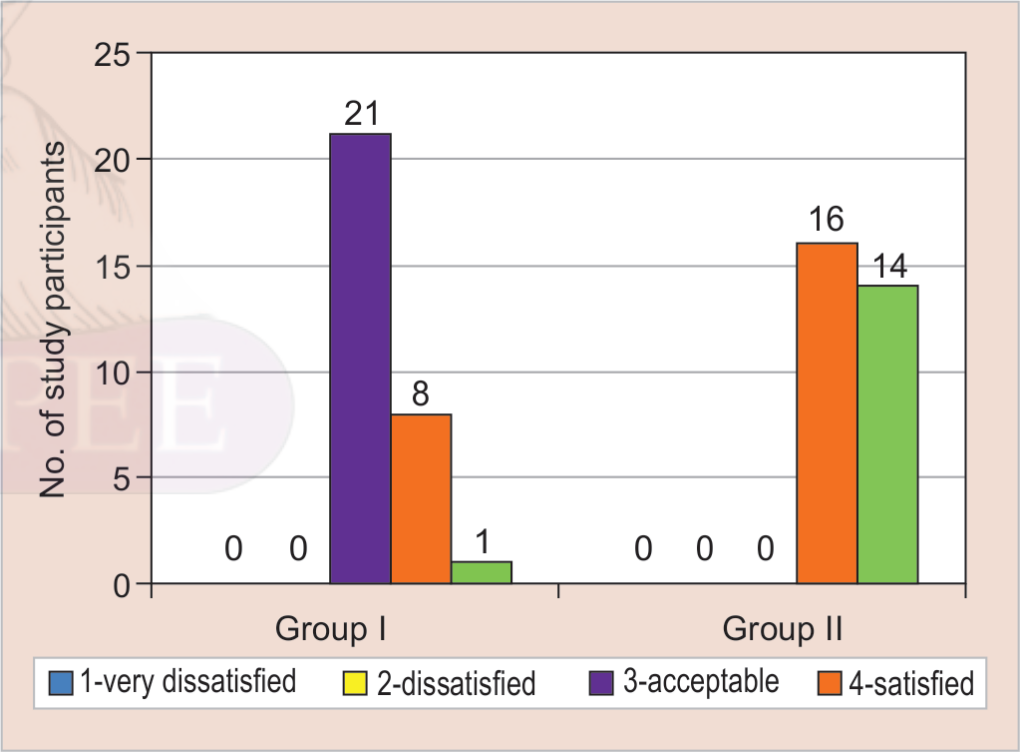
Graph showing the parental perception about change in behavior of the child towards appearance of the restoration for the conventional composite material in control group (group A) and colored compomer material in experimental group (group B)

On applying the chi-square test, both the groups had a significant response regarding parental perception about change in the behavior of the child towards dental treatment, appearance, and color of the restoration (Graph 2). However, group B (experimental group) reported a higher score for parental perception about change in the behavior of the child towards dental treatment, color, and appearance of the restoration (Graphs 3 and 4).

**Fig. 1A:**
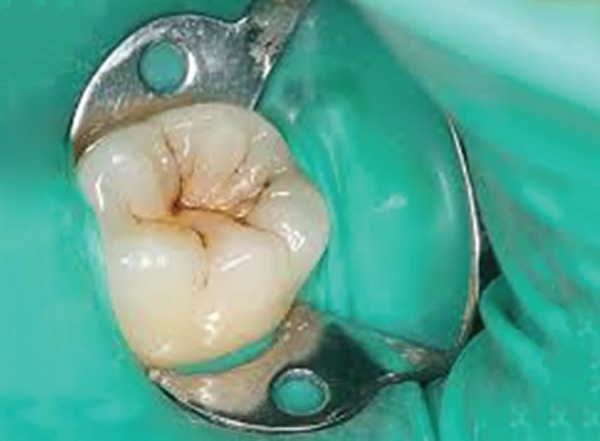
Preoperative photograph showing selected teeth for the conventional composite material in control group (group A)

**Fig. 1B:**
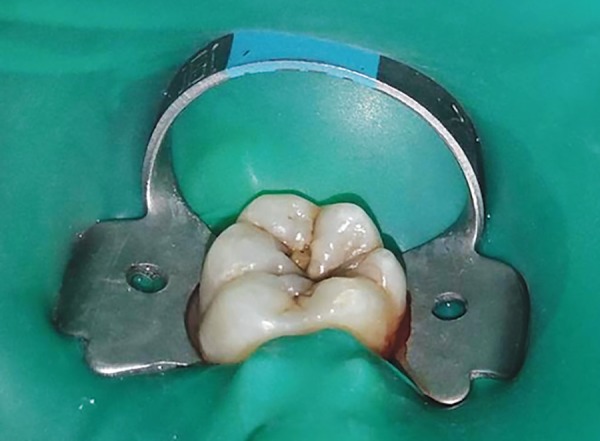
Preoperative photograph showing selected teeth for colored compomer material in experimental group (group B)

**Fig. 1C:**
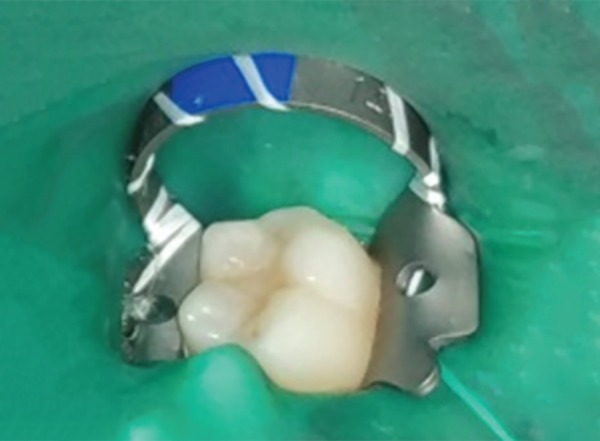
Postoperative photograph showing teeth restored with conventional composite material in control group (group A)

**Fig. 1D:**
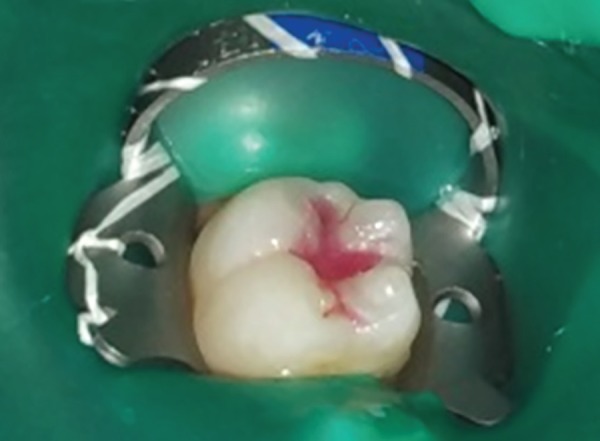
Postoperative photograph showing teeth restored with colored compomer material in experimental group (group B)

**Fig. 1E:**
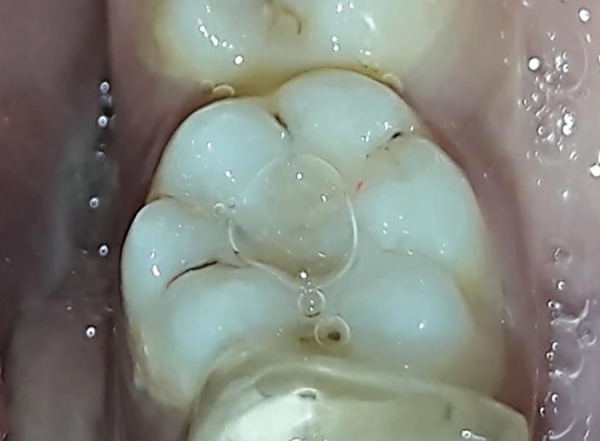
Postoperative photograph showing teeth restored with conventional composite material in control group (group A) after 6 months follow-up

**Fig. 1F:**
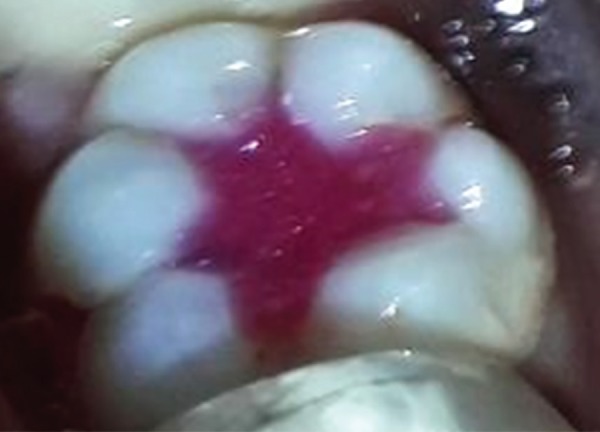
Postoperative photograph showing teeth restored with colored compomer material in experimental group (group B) after 6 month follow-up

All 60 restorations were followed-up for 6 months interval for clinical success. No statistical differences were established between both the groups at the end of the first month (Table 2).

At 3 months follow-up, both the groups showed a clinically insignificant difference for all the parameters but marginal discoloration was highly statistically significant (*p* = 0.005) (Table 3).

**Table 1:** Preoperative and postoperative comparison of anxiety teeth for the conventional composite material in control group (group A) and colored compomer material in experimental group (group B)

*Paired samples statistics*					
*Groups*	*Mean*	*Std. deviation*	*Std. error mean*	*t*	*Sig. (2-tailed)*
A	2.05	1.067	0.087	−8.464	0.000
	3.00	1.264	0.103		
B	1.93	1.060	0.087	−13.259	0.000
	3.51	1.360	0.111		

At 6 months, 13.3% change was seen in the marginal integrity of composite restorations as compared to 10.0% of compomer restorations and 6.7% change in anatomic form for composites as compared to 13.3% of compomer. The change in secondary caries was 10.0% in the composite group as compared to no change in compomer group. Marginal discoloration was observed in 23.3% of subjects for compomers. Among both the materials there was no statistically significant difference, but marginal discoloration was highly significant (*p* = 0.005) (Table 4).

## DISCUSSION

Dentistry for the children requires a basic restorative material which is simple to use, have good adhesive properties and good clinical longevity. In recent times, esthetic materials like composites and compomers are used for both primary and permanent anterior and posterior teeths.

**Table 2:** Retention rates for conventional composite material in control group (group A) and colored compomer in experimental group (group B) at one month

*One Month*									
*Criteria*	*Group A*				*Group B*				*Chi-square test*
	*Score*				*Score*				
	*1*	*2*	*3*	*4*	*1*	*2*	*3*	*4*	
Marginal integrity	100%	100%	0%	0%	100%	100%	0%	0%	No statistics computed
Anatomic form	100%	100%	0%	0%	100%	100%	0%	0%	
Secondary caries	100%	100%	0%	0%	100%	100%	0%	0%	
Marginal discoloration	100%	100%	0%	0%	100%	100%	0%	0%	

**Table 3:** Retention rates for conventional composite material in control group (group A) and colored compomer in experimental group (group B) at three months

*Criteria*	*Three months*								*Chi-square test*
	*Group A*				*Group B*				
	*Score*				*Score*				
	*1*	*2*	*3*	*4*	*1*	*2*	*3*	*4*	
Marginal integrity	100%	100%	0%	0%	100%	100%	0%	0%	No statistics computed
Anatomic form	100%	100%	0%	0%	93.3%	6.7%	0%	0%	*p* = 0.150 X^2^ = 2.069 df = 1
Secondary caries	93.3%	6.7%	0%	0%	100%	100%	0%	0%	*p* = 0.150 X^2^ = 2.069 df = 1
Marginal discoloration	100%	100%	0%	0%	76.7%	23.3%	0%	0%	*p* = 0.005 X^2^ = 7.925 df = 1

**Table 4:** Retention rates for conventional composite material in control group (group A) and colored compomer in experimental group (group B) at six months

*Criteria*	*Six Month*								*Chi-square test*
	*Group A*				*Group B*				
	*Score*				*Score*				
	*1*	*2*	*3*	*4*	*1*	*2*	*3*	*4*	
Marginal integrity	86.7%	13.3%	0%	0%	90.0%	10.0%	0%	0%	*p* = 0.688 X_2_ = 0.162 df = 1
Anatomic form	93.3%	6.7%	0%	0%	86.7%	13.3%	0%	0%	*p* =0.389 X_2_ = 0.741 df = 1
Secondary caries	90.0%	10.0%	0%	0%	100%	100%	0%	0%	*p* = 0.675 X_2_ = 0.756 df = 1
									
									
Marginal discoloration	100%	100%	0%	0%	76.7%	23.3%	0%	0%	*p* = 0.005 X_2_ = 7.925 df = 1

Composite (componere = to combine) is the commonly used basic direct tooth-colored restorative material because of their characteristics like esthetics, good adhesive technology, and superior properties when compared to amalgam.^6^ Composites have certain disadvantages like polymerization shrinkage which results in volumetric contraction, cusp deformation, microcracks in enamel, reduced marginal adaptation, and sensitivity after restorations.

Compomers came into existence in 1992, and it has the mechanical and esthetic properties of composites and glass-ionomer restorative materials.^7^ Compomers have shown lesser thermal conductivity, conservation of tooth structure, dimensionally stable, fluoride release which makes more acceptable to children and parents.^8^

Sigmund Freud theory states that there is a development of egocentricity in growing up children. Thus, children prefer selecting the things they like, be it their clothes or the color of their restoration. Being pediatric dentists, it is of utmost importance to evaluate if there is any change in clinical success of multicolored restoration and conventional materials.

In the last decade, a new colored compomer material was introduced into the market. This material will be accepted by children because of its attractive colors. Since there was less literature available on the restorative material, the present study was carried out to evaluate and compare the clinical success rate of the colored compomer and composite in occlusal caries of permanent molar teeth. In our study, the split-mouth design helped to overcome the patient bias.

A child's behavior and psychology can be assessed by his or her clothes, toys, and home accessories through colors. The present study stated a reduction in the dental anxiety in compomer group because specific color elicits specific emotional responses.^9^ The children, when given chance to select the restoration colors, are showing more positive, and also evaluation will be good if the particular restoration is missing.^10^

There were no statistically significant differences among both the groups at 1, 3 and 6 months follow-up when assessed for clinical parameters like marginal integrity, anatomic form, secondary caries and surface texture except for marginal discoloration (*p* = 0.005). The marginal discoloration seen was nothing but the loss of glitter particles at the edges of the restoration.

Compomer are very helpful in children because it can be easily manipulated, its consistency makes it easy to use, placed in cavity without sticking, also it requires less time for final polishing.^11,12^ The past experiments have shown that compomers have better clinical success rates so can be considered as suitable alternative to amalgam in children.^13–15^ Dyract restorations have shown 100% retention which was in accordance with our study because of standardization followed in the clinical study.^16^

In our study, the margins showed discoloration with loss of glitter particles, but there was no secondary caries it may be due to minimal mechanical retention in adhesive cavities, technique problems as discussed by Andersson- Wenckert et al.^17^ and Peters et al.^18^ The compomer material showed no secondary caries when compared to composites (6%) in other studies.^19,20^ This study has shown that multicolored compomer may be used as an alternative to conventional compomers because of its high clinical success rate showed after 6 months follow-up. The limitations of this study were a smaller sample size with an only 6 months follow-up period. In future, a larger sample size with long follow-up period is required to prove its clinical longevity.

## CONCLUSION

Both composites and multicolored compomer materials have shown promising results in the treatment of occlusal caries of permanent teeth but the multicolored compomer material has shown promising results and it permits the child to settle on the color of the filling makes them participate in the treatment and decreases the anxiety of the child and improves the child behavior. Thus multicolored restorations act as positive reinforcement in behavioral management of children.

## CLINICAL SIGNIFICANCE

Colored compomers are known to be excellent alternative restorative materials for restoration of teeth in children as they aid in behavior modification and good compliance from the patient.

## MANUFACTURER

- Composite (3M ESPE, St. Paul, Minn., USA)
- Coloured Compomer (Twinky star, Voco, Cuxhaven, Germany)
- Bonding system (Futurabond NR, Voco)
